# Retrospective Cohort Study of Early versus Delayed Ballon Kyphoplasty Intervention for Osteoporotic Vertebral Fracture Treatment

**DOI:** 10.3390/medicina60040519

**Published:** 2024-03-22

**Authors:** Akiyoshi Miyamoto, Umesh Parihar, Chetan Kumawat, Abd El Kader Al Askar, Masato Tanaka, Sharvari Gunjotikar, Takuya Taoka, Tadashi Komatsubara, Yoshihiro Fujiwara, Koji Uotani, Shinya Arataki

**Affiliations:** 1 Department of Orthopaedic Surgery, Okayama Rosai Hospital, Okayama 702-8055, Japan; akkun@kzd.biglobe.ne.jp (A.M.); gtbumesh@gmail.com (U.P.); dr.ckumawat@gmail.com (C.K.); khattab2013@gmail.com (A.E.K.A.A.); sharvarigunjotikar@gmail.com (S.G.); taokatakuya@gmail.com (T.T.); t.komatsubara1982@gmail.com (T.K.); fujiwarayoshihiro2004@yahoo.co.jp (Y.F.); araoyc@gmail.com (S.A.); 2 Department of Orthopaedic Surgery, Okayama University Hospital, Okayama 7000-8558, Japan; coji.uo@gmail.com

**Keywords:** ballon kyphoplasty, osteoporotic vertebral fractures, kyphosis

## Abstract

*Objectives*: To investigate the outcomes of early balloon kyphoplasty (BKP) intervention compared with late intervention for osteoporotic vertebral fracture (OVF). *Background*: Osteoporotic vertebral fracture can lead to kyphotic deformity, severe back pain, depression, and disturbances in activities of daily living (ADL). Balloon kyphoplasty has been widely utilized to treat symptomatic OVFs and has proven to be a very effective surgical option for this condition. Furthermore, BKP is relatively a safe and effective method due to its reduced acrylic cement leakage and greater kyphosis correction. *Materials and Methods*: A retrospective cohort study was conducted at our hospital for patients who underwent BKP for osteoporotic vertebral fractures in the time frame between January 2020 and December 2022. Ninety-nine patients were included in this study, and they were classified into two groups: in total, 36 patients underwent early BKP intervention (EI) at <4 weeks, and 63 patients underwent late BKP intervention (LI) at ≥4 weeks. We performed a clinical, radiological and statistical comparative evaluation for the both groups with a mean follow-up of one year. *Results*: Adjacent segmental fractures were more frequently observed in the LI group compared to the EI group (33.3% vs. 13.9%, *p* = 0.034). There was a significant improvement in postoperative vertebral angles in both groups (*p* = 0.036). The cement volume injected was 7.42 mL in the EI, compared with 6.3 mL in the LI (*p* = 0.007). The mean surgery time was shorter in the EI, at 30.2 min, compared with 37.1 min for the LI, presenting a significant difference (*p* = 0.0004). There was no statistical difference in the pain visual analog scale (VAS) between the two groups (*p* = 0.711), and there was no statistical difference in cement leakage (*p* = 0.192). *Conclusions/Level of Evidence*: Early BKP for OVF treatment may achieve better outcomes and fewer adjacent segmental fractures than delayed intervention.

## 1. Introduction

Our bones, the framework that supports our body, undergo a constant process of renewal. Osteoporosis disrupts this delicate balance, leading to a decrease in bone mineral density and making bones more susceptible to fractures. According to the World Health Organization (WHO) 1994 definition, osteoporosis is the most widespread bone disorder globally, characterized by a T-score of −2.5 standard deviations (SDs) or more below the bone mineral density (BMD) of a healthy individual of the same sex at a younger age [1]. Osteoporosis is increasing dramatically with the aging of the global population [2]. Osteoporosis-related fractures, such as femoral neck fractures, distal radius fractures and vertebral fractures, are primary concerns for the elderly [3]. Furthermore, these fractures may increase the morbidity and mortality risks among osteoporotic patients [4].

Osteoporotic vertebral fractures (OVFs), the most common osteoporosis-related fractures, can lead to kyphotic deformity, severe back pain, depression and disturbances in activities of daily living (ADL) [5]. Traditional treatments for OVFs often focus on pain management and preventing further fractures [6]. These treatments include medication, physical therapy and bracing. While these approaches can be helpful, they do not address the underlying fracture itself. Cement-injection techniques like vertebroplasty and kyphoplasty are potential solutions for OVFs to stabilize them and reduce pain. Percutaneous balloon kyphoplasty (BKP) has been widely utilized to treat symptomatic OVFs and has proven to be a very effective surgical option for this condition [7]. Compared with vertebroplasty, BKP is a relatively safe and effective method due to its reduced acrylic cement leakage and greater kyphosis correction [8,9].

According to current guidelines, patients are primarily recommended to undergo conservative treatment for osteoporotic vertebral fractures. Surgical intervention is recommended when conservative treatments are ineffective clinically or if there is radiographic progression of the fracture [10]. However, prolonged bed rest in the elderly may lead to increased mortality rates and complications such as pneumonia, myocardial infarction/cardiac complications, deep venous thrombosis (DVT) and urinary tract infection [11]. Determining the appropriate duration of conservative therapy is crucial for symptomatic osteoporotic vertebral fractures to achieve clinical benefit [12]. Several reports discuss the timing of balloon kyphoplasty [12,13,14,15]. Fracture reduction was best achieved in acute fractures [12,13]. Palmowski recommended making a final decision about conservative vs. operative treatment within 6 weeks to ensure better height restoration in surgically treated patients [14]. However, early intervention may create a higher risk of cement leakage and cement leakage is ta risk factor for adjacent segment fracture [15].

Current guidelines lack consensual recommendations for the timing of percutaneous balloon kyphoplasty (BKP) [16,17]. Therefore, we performed a retrospective comparative cohort study to save more time and decrease the burden on future research funding. The aims of this study were to investigate the clinical and radiographic outcomes, as well as complications, associated with the early intervention of PKP to provide current evidence for spine surgeons.

## 2. Materials and Methods

**Figure 1:**
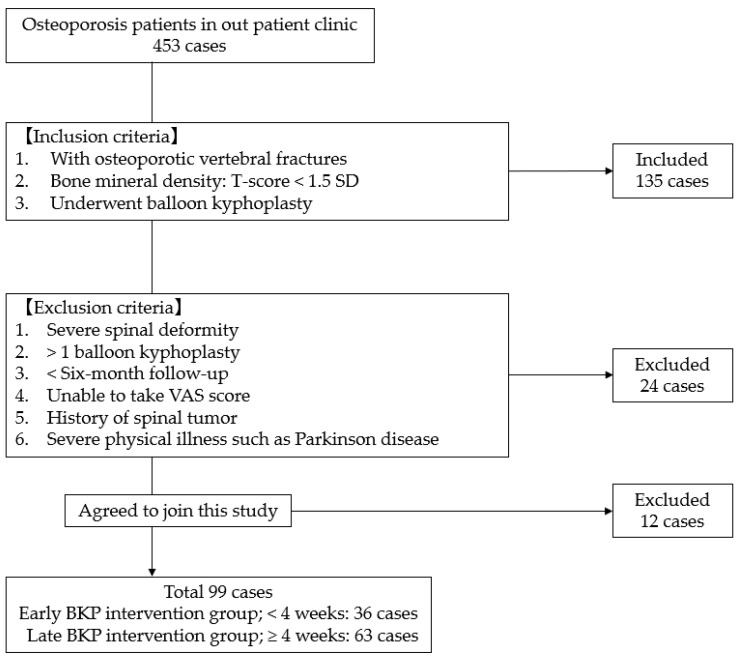
Patient selection.

This research was approved by the ethics committee of our institution (Okayama Rosai Hospital, 2023, No. 472), and informed consent from patients undergoing surgery was duly obtained. A retrospective evaluation was conducted for patients who underwent percutaneous balloon kyphoplasty (PKP) for osteoporotic vertebral fractures (OVFs) at our hospital in the time frame between January 2020 and December 2022. The inclusion criteria for this research were as follows: (1) Presence of osteoporotic vertebral fractures, (2) bone mineral density T-score < 1.5 SD, (3) patient underwent balloon kyphoplasty. Exclusion criteria included: (1) Severe spinal deformity (scoliosis, sagittal vertical axis > 10 cm), (2) >1 level balloon kyphoplasty, (3) <six months of follow-up, (4) inability to provide valid VAS scores, (5) history of spinal tumor, (6) severe physical illness, such as Parkinson;s disease (Figure 1).

Between January 2020 and December 2022, there were 453 patients in our outpatient clinic. In total, 135 patients were matched with the inclusion criteria. Furthermore, 24 patients were excluded by the exclusion criteria and 12 patients did not agree to take part in this study. In total, 99 patients were included in this study (Figure 1). Thirty-six patients underwent early BKP interventions (Group EI) < 4 weeks, and 63 patients underwent late BKP interventions (Group LI) ≥ 4 week.

### 2.1. Clinical Evaluation

The cohort of patients underwent clinical assessment using standard quantifiable measures, including the visual analogue scale (VAS) for low back pain and the Oswestry Disability Index (ODI) before and after surgery. The ODI is used to assess how a patient’s low back pain affects their ability to perform everyday tasks and activities. Clinical data were documented preoperatively, postoperatively and at final follow-up time periods. Surgical duration and any complications, such as sensory motor deficits, surgical site infections and the need for revision surgery, were noted during the intraoperative or postoperative periods. The visual analog scale (VAS) for back pain was used to assess clinical outcomes.

### 2.2. Radiological Evaluation

**Figure 2:**
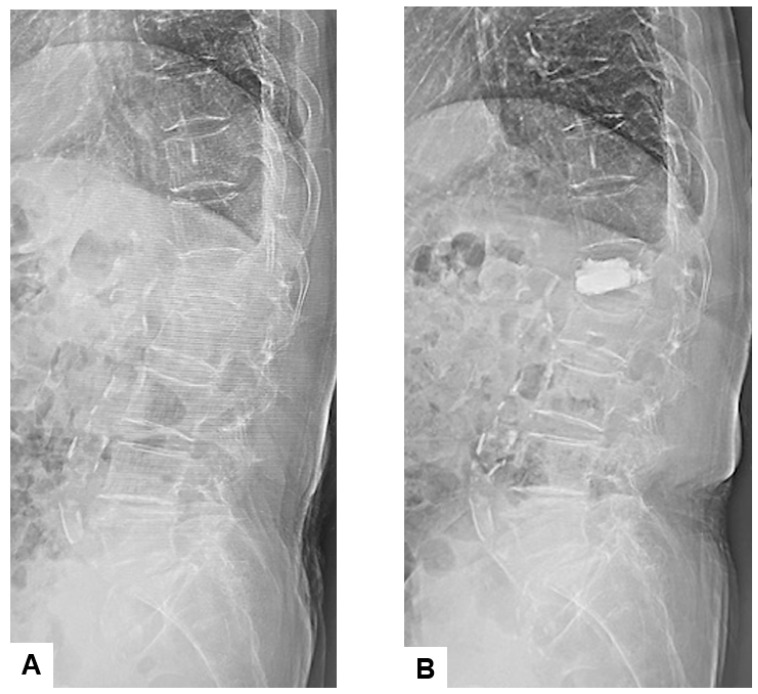
Standing lumbar lateral radiograms: (**A**) preoperative, (**B**) postoperative. The parameters such as the local kyphosis angle (LKA), vertebral kyphotic angle (VKA), cement leakage and adjacent vertebral fractures (AVFs) were calculated.

For radiological evaluation, parameters such as the local kyphosis angle (LKA), vertebral kyphotic angle (VKA), cement leakage and adjacent vertebral fractures (AVFs) were calculated preoperatively and postoperatively, with standing plain radiograms if possible (Figure 2).

Additionally, preoperative bone mineral density (BMD) T-score and fracture level were evaluated. The BMD was mainly taken after fracture with the same software and equipment in our hospital. The T-score was taken from lumbar spine from L2–5. If the fractured vertebrae or severe deformed vertebrae were included in this area, we excluded these vertebrae.

### 2.3. Statistical Evaluation

All collected data were expressed as means ± standard deviations (SDs). The Shapiro–Wilk normality test histogram for the values was used to check normal distribution. The result was not a normal distribution. In comparing the groups, the Mann–Whitney U test analysis was used for continuous variables, while the chi-squared test and Fisher’s exact test were used for dichotomous variables. McNemar’s test was used to compare the *p*-values. A *p*-value < 0.05 was defined as statistically significant. All analyses were performed using SPSS version 19.0 (IBM, Beijing, China).

### 2.4. Surgery

**Figure 3:**
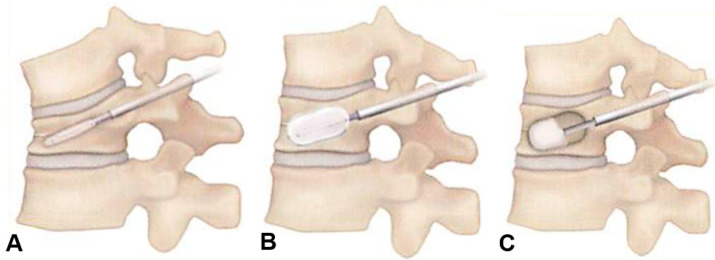
**Surgical procedure,** (**A**): Small balloons are guided through each cannula into the vertebra. (**B**): Each balloon is carefully inflated to raise the collapsed vertebra. (**C**): The balloons are deflated and removed. The resultant cavities are filled with bone cement.

All procedures were conducted using standardized kyphoplasty (Kyphon Ballon Kyphoplasty System, Medtronic, Minneapolis, MN, USA). Percutaneous balloon kyphoplasty (BKP) was performed using a bilateral inflatable balloon tamp via a bilateral transpedicular approach. After general anesthesia, the patient was carefully positioned prone on the operating table. Subcutaneous local 1% lidocaine was administered at the planned needle entry site for the both pedicles, which was localized by C-arm. A small, 4-millimeter skin incision was made and an 11-guage needle was advanced through the both pedicle into the vertebral body utilizing intermittent C-arm. Special attention was paid to performing a medial trajectory of the needle for a final midline destination of the needle tip in the vertebral body. The needle was enlarged with a working cannula over a guide wire, through which a drill was advanced, creating a channel for the balloon. Next, the inflatable balloon tamp was advanced into the anterior third of the vertebral body under C-arm. The balloon was inflated under C-arm at less than 200 psi and about 4 mL of contrast medium. The balloon was deflated and removed. Polymethylmethacrylate bone cement was prepared and inserted with a special cannula and an inner instrument into the cavity under C-arm (Figure 3). The amount of bone cement should be approximately the same volume as balloon expansion; however, the most important point was to prevent cement leakage. The vertebral body cavity was filled with bone cement to prevent bone cement from backing out from the cannula with the inner instrument. Finally, all instrumentation was removed at the end of the procedure. There was no restriction for the patient and the patient reported immediate pain relief. The procedure was performed by the same surgical team. Each of four doctors was an experienced spine doctor and able to perform this BKP procedure.

## 3. Results

### 3.1. Patient Demographics

Ninety-nine patients were included in the study after meeting the criteria, and they were divided into two groups: an early intervention group (Group EI, <4 weeks) and a late intervention group (Group LI, ≥4 weeks). Group EI consisted of 11 male and 25 female patients with an average age of 81.8 ± 5.0 years, while Group LI consisted of 25 male and 38 female patients with an average age of 80.1 ± 8.2 years. The mean follow-up period for Group EI was 11.2 ± 7.4 months, while for Group LI, it was 12.1 ± 9.4 months. The patients’ demographics are summarized in Table 1. There were no differences in age at surgery, sex, or follow-up period between the two groups.

### 3.2. Clinical Results

The body mass indices (BMIs) of the both groups presented no differences. The surgery duration for Group EI was 30.2 ± 7.4 min, a little shorter than that of Group LI (37.1 ± 8.9 min, *p* = 0.0004). The final VAS results for low back pain in both groups presented no differences (11.3 ± 10.9 vs. 13.5 ± 13.2, *p* = 0.722). Revision surgery was performed on three patients in Group EI and four patients in Group LI (8.3% vs. 6.3%, *p =* 0.711) (Table 2).

### 3.3. Radiographic Results

**Figure 4:**
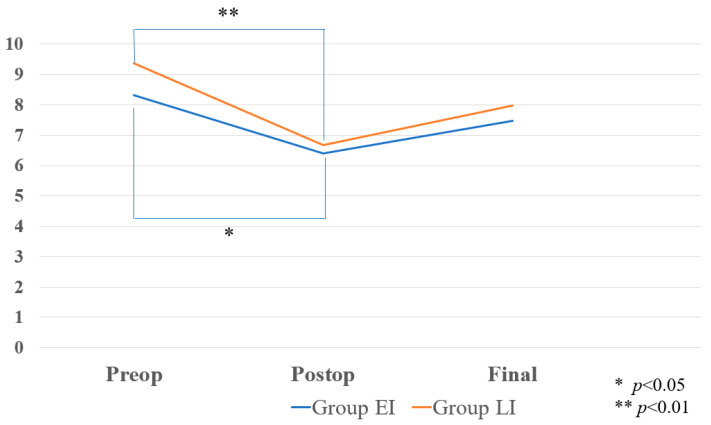
Chronological results of vertebral kyphosis angle.

**Figure 5:**
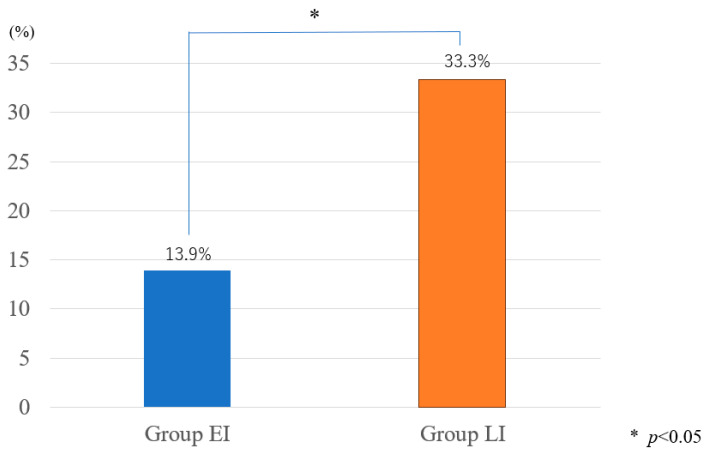
Adjacent segmental fractures.

There was a significant improvement in the postoperative vertebral angles (VKA) in both groups (Figure 4: *p* = 0.036, 0.00027). Adjacent vertebral fractures (AVFs) were observed with greater frequency in the LI group than in the EI group, with rates of 33.3% for the LI group compared to 13.9% for the EI group, as shown in Figure 5. This difference, marked by a *p*-value of 0.034, indicates statistical significance, suggesting a higher risk of AVFs following late intervention. On the other hand, the incidence of cement leakage during the procedures was noted to be higher in the EI group compared to the LI group, with respective rates of 30.1% and 19.0%. Despite this observation, the difference in the cement leakage rates between the two groups did not reach statistical significance, as reflected by the *p*-value of 0.192.

**Figure 6:**
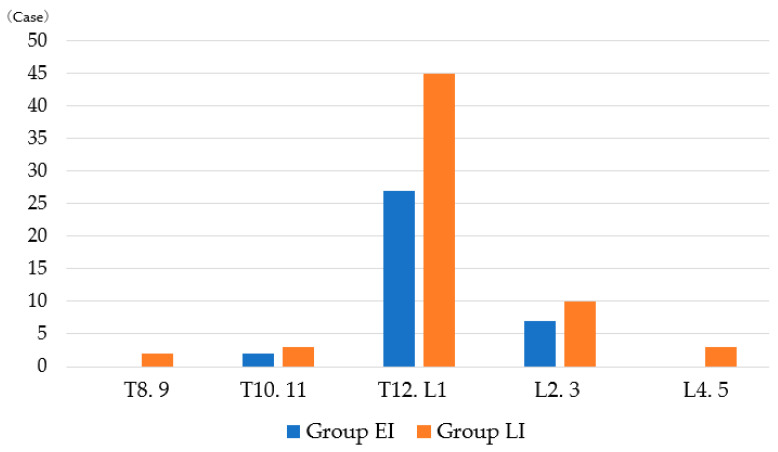
Number of fractures per site.

The ranking of the fracture sites is summarized in Figure 6. The thoracolumbar area was the most common site of osteoporotic vertebral fractures in both groups. In group EI, there were two in the thoracic subgroup (T8–11), 27 (75.0%) in the thoracolumbar subgroup (L1, T12) and seven in the lumbar subgroup (L2–4). In Group LI, there were five in the thoracic subgroup, 45 (71.4%) in the thoracolumbar subgroup and 13 in the lumbar subgroup.

The radiographic results are summarized in Table 3. In Group EI, the preoperative, postoperative and final local kyphosis angles (LKAs) were 8.31 degrees, 6.41 degrees and 7.47 degrees, respectively. In Group LI, the preoperative, postoperative and final LKAs were 9.37 degrees, 6.68 degrees and 7.97 degrees, respectively. There was no statistically significant difference between the two groups. For the group receiving early intervention, identified as group EI, the sequence of measurements for the local kyphosis angles (LKAs) commenced with a preoperative average of 8.31 degrees. This initial measurement underwent a decrease postoperatively to an average of 6.41 degrees, showcasing the effect of the intervention. By the time of the final follow-up, the average LKA in this group had slightly increased to 7.47 degrees, indicating a degree of relapse, but still demonstrating an improvement over the preoperative state. Meanwhile, the group subjected to late intervention, Group LI, started with a slightly higher preoperative LKA average of 9.37 degrees. Following the intervention, this measurement saw a reduction to an average of 6.68 degrees postoperatively, similar to the improvement seen in Group EI. The final average LKA in Group LI settled at 7.97 degrees, reflecting a comparable final outcome to that of Group EI, albeit slightly higher. Despite these changes and improvements in both groups, the statistical analysis revealed that the differences between the groups in terms of changes in local kyphosis angles from before surgery to after and, ultimately, to the final measurement point were not statistically significant.

Moreover, within the early intervention cohort, denoted as Group EI, the initial vertebral kyphotic angles (VKAs) prior to surgery were recorded at an average of 14.1 degrees. Following the intervention, these VKAs showed a notable decrease, averaging 6.5 degrees, indicative of substantial post-surgical improvement. At the point of the final evaluation, the VKAs in this group had adjusted to an average of 11.1 degrees, highlighting a regression from the postoperative state but still marking an improvement on the preoperative condition. Conversely, the late intervention cohort, referred to as Group LI, exhibited pre-surgical VKAs that averaged 14.9 degrees, a figure that was slightly higher compared to Group EI. Post-surgery, the average VKA in Group LI was reduced to 7.7 degrees, illustrating a significant reduction, albeit starting from a marginally more severe baseline condition. By the time of the final assessment, the VKAs for Group LI averaged 10.4 degrees, demonstrating a slightly more favorable outcome in terms of angle reduction compared to Group EI, despite the marginally worse initial condition. The statistical analysis yielded *p*-values of 0.35, 0.67 and 0.53, respectively, for the comparison of the VKA changes from the preoperative to the postoperative and to the final follow-up measurements between the two intervention groups, signifying that the differences in the vertebral kyphosis angle adjustments observed from the initial state through to the final follow-up between the early and late intervention groups did not achieve statistical significance. The bone mineral densities (BMDs) (T-score) for Group EI and Group LI were −2.1 and 1.6, respectively (*p* = 0.10), which was statistically insignificant.

## 4. Discussion

Osteoporotic vertebral fracture (OVF) stands as the most prevalent fragility fracture. Studies indicate that about 25% of women aged over 70 years and over half of those aged above 80 years experience at least one OVF during their lifetime [18]. The elderly population most commonly experiences OVFs, resulting in significant pain and a notable decline in quality of life [19,20]. Conservative management, as well as percutaneous vertebroplasty (PVP), balloon kyphoplasty (BKP) and vertebral body stenting (VBS), is employed for their treatment [21]. Several factors drive the preference for conservative management as the initial choice for osteoporotic vertebral fractures (OVFs). Delayed diagnosis due to subtle symptoms and a lack of awareness about OVF complications lead to the underestimation of the condition’s severity. Economic constraints limit access to specialized care and surgery, making conservative options like pain management and physical therapy more feasible and accessible for many patients. This leads to delays in seeking vertebral body augmentation interventions, which can promptly alleviate pain and enhance functionality [11,22,23]. The mortality rate rises significantly in patients with OVF and this risk escalates with the number of affected vertebrae. Furthermore, for each diagnosed OVF in a patient, the likelihood of subsequent fractures increases by a factor of five [24]. Despite adjusting for demographic characteristics and chronic comorbidities, the risk of mortality was 1.22 times higher for the OVF [25].

It is conceivable that fracture consolidation could restrict the possible height restoration if there is a lengthy period between the fracture and surgery. The application of BKP is safe and helpful for patients with acute OVF and ongoing discomfort. Following a percutaneous vertebroplasty, patients have rapid, long-lasting pain reduction that is affordable and far more effective than what would be possible with conservative care [2]. Important characteristics that set balloon kyphoplasty apart from percutaneous vertebroplasty are height restoration and decreased cement leakage [26]. Overall procedural complication incidents with vertebroplasty have been reported at 2.4% [27], compared to 0.9% incidents with kyphoplasty [9]. The best time to execute a balloon kyphoplasty has not been sufficiently investigated [9]. Some studies suggest a conservative treatment effort lasting two to three weeks, while others offer no advice at all about time [28,29]. Based on the provided demographic data and follow-up periods, our study revealed that the demographic characteristics of the two groups, early intervention (Group EI) and late intervention (Group LI), were quite similar. Both groups comprised a comparable distribution of male and female patients with similar mean ages (81.8 ± 5.0 years for Group EI and 80.1 ± 8.2 years for Group LI). Additionally, the mean follow-up periods for both groups were also comparable (11.2 ± 7.4 months for Group EI and 12.1 ± 9.4 months for Group LI), indicating a balanced representation and duration of observation. Furthermore, it is noteworthy that the bone mineral densities (BMDs) in Group EI and Group LI were also found to be similar, with T-scores of −2.1 for Group EI and 1.6 for Group LI, although this difference was statistically insignificant (*p* = 0.10).

In our study, both the early intervention (EI) and the late intervention (LI) groups demonstrated dramatic improvements in visual analogue scale (VAS) scores at the final follow-up. However, no statistical differences were observed in the final VAS scores between the EI and LI groups. This finding aligns with previous studies comparing outcomes based on different surgical timings of cement augmentation, where similar improvements in VAS scores were reported regardless of whether the operation was performed within 4 weeks or later [17,30]. These consistent improvements in VAS scores suggest that the timing of BKP may not significantly affect pain outcomes in patients with OVFs. The similarity between the pain outcomes in the early and late surgical intervention groups may have been due to cement augmentation stabilizing micromovements and disrupting nerve endings, regardless of timing [31,32].

In terms of surgery durations, we found a notable difference between the EI (early intervention) and LI (late intervention) groups. The mean surgery duration for Group EI was reported to be 30.2 ± 7.4 min, whereas it was 37.1 ± 8.9 min for Group LI [33]. Our findings were supported by Li et al.’s 2015 study [33]. Early intervention may involve less complex procedures or fewer technical challenges due to the relatively intact structural integrity of the vertebral body, resulting in shorter surgical durations. In contrast, late intervention often occurs after significant vertebral collapse or deformity has occurred, necessitating more intricate surgical techniques and potentially prolonging the duration of the procedure. Thoracolumbar area was the most common site of fracture for both the groups in the present study, which was consistent with the results of a study by Oh et al. [9]. Our results demonstrated a significantly higher incidence of AVF in the LI group compared to the EI group (13.9% vs. 33.3%, *p* = 0.034). This finding suggests a potential association between delayed surgical intervention and an increased risk of adjacent segmental fractures, highlighting the importance of timely BKP in preventing such complications.

Our findings are consistent with previous retrospective studies that have investigated the impact of surgical timing on the risk of subsequent fractures following PKP or vertebroplasty [17,34,35]. The underlying mechanism of the vertebral recollapse remains unclear. The strain on neighboring vertebrae is increased by the significant kyphotic deformity of delayed BKP. Furthermore, AVFs have been linked to the abnormal sagittal alignment of the vertebral bodies. In LI, endplate damage can result from repeated compressive pressure over the fracture site, which can cause bone loss close to the endplate. Repeated damage during everyday activities can cause a void to form above the LI fracture site. The incidence of AVFs may rise because of the impact of this void, which may also lessen the intergradient effect between the cement and the bone. Extended bed rest in LI negatively affects back muscles, leading to a loss of muscle mass, which reduces vertebral body protection and increases the risk of further compression fractures [12]. This phenomenon is likely to be associated with changes in the local biomechanical environment that arise from the kyphotic collapse of vertebral compression fractures [36].

The most frequent complication of kyphoplasty is the leakage of bone cement, which can pose significant risks to patients, even when present in minimal amounts [7]. At the upper end of the spectrum, radiculopathy rates of 4% and cord compression rates of less than 0.5% have been reported [37]. Our analysis indicated that the rates of cement leakage were comparable between the early intervention (EI) group and the late intervention (LI) group, recorded at 30.1% and 19.0%, respectively. The observed difference, with a *p*-value of 0.192, did not reach statistical significance. This suggests that the timing of the intervention, whether earlier or later, does not significantly affect the likelihood of cement leakage during the procedure. Our results showed that the cement leakage in the EI group compared to the LI group was not statistically significant. The cement leakage rates in both groups in this study were relatively high compared with a previous report [11]. This was mainly because cement leakage was evaluated by postoperative radiograms and CT in this study. Interestingly, the findings from another study, by Huaquing Guan et al. [30], suggest that delayed operations may reduce the incidence of cement leaks. The rate of polymethylmethacrylate leakage is a subject of controversy and seems to be somewhat technique-dependent [38]; alternatively, factors such as the pressure, volume and viscosity of the cement or technique used may play a more direct role in determining the risk of these types of leakage, rather than the timing of the surgical intervention [11]. One study suggests that reducing the cement-injection volume could potentially decrease cement leakage [39]. By contrast, our study did not find a significant correlation between the clinical outcomes and the volume of cement injected. We found that more cement could be injected in the early group. The EI group had an average cement volume of 7.42 ± 1.54 mL, while the LI group used a lesser amount, averaging at 6.3 ± 1.93 mL. This variation was statistically significant, with a *p*-value of 0.007, indicating that the amount of cement utilized in the procedure varied notably with the timing of the intervention. A larger retrospective study also did not find a significant association between the injected cement volume and post-procedural pain [13], but the volume of injected cement was predicted to be a factor in pain release in another study [40].

Both the EI group and the LI group in our study exhibited improvements in VKA from the preoperative to the final measurements. In our study, the VKA in the final follow-up in the early group was slightly better than that in the late group, but there was no significant difference. In Group EI, the preoperative, postoperative and final VKAs were 14.1 degrees, 6.5 degrees and 11.1 degrees, respectively. In Group LI, the preoperative, postoperative and final VKAs were 14.9 degrees, 7.7 degrees and 10.4 degrees, respectively (*p* = 0.53). Other studies have demonstrated a notable improvement in VKA levels within the early-treatment group compared to the late group [5,17]. The VKA of final follow-up showed an increased angle mainly because the remaining cancellous bone was collapsed gradually. Similarly to our study, the enhancement observed was not statistically significant in a different study [7].

It is important to acknowledge the limitations of this study. As a retrospective study, its findings are subject to inherent biases. The fractured vertebrae were not equally distributed in the two groups. The short follow-up and the relatively small sample size further constrain the generalizability of the results. Future research should aim to mitigate these limitations by incorporating larger, more diverse cohorts and employing a prospective study design where feasible. A longer follow-up duration would be instrumental in assessing the sustainability of treatment effects and the incidence of long-term complications, such as adjacent vertebral fractures or the need for subsequent interventions. Furthermore, exploring the biomechanics of adjacent fractures could offer valuable insights into prevention strategies and refine surgical techniques to minimize the risk of secondary fractures. An analysis focused on the potential influence of gender, as well as other demographic and clinical variables, on treatment outcomes could facilitate more personalized approaches to managing OVFs. Moreover, a deeper understanding of patient preferences, barriers to early intervention and the impact of socioeconomic factors on treatment choices would foster more comprehensive approaches to care.

## 5. Conclusions

In conclusion, early BKP may achieve better clinical and radiographic outcomes for treating osteoporotic vertebral fractures than delayed BKP. Early-intervention BKP leads to more efficient surgical outcomes, including shorter operative times and a reduced risk of subsequent vertebral fractures. Based on current evidence, early BKP intervention might be more beneficial to patients.

## Figures and Tables

**Table 1:** Patient demographic.

	Group EI	Group LI	*p* Value
Number	36	63	
Age at surgery (year)	81.8 ± 5.0	80.1 ± 8.2	0.338
Sex (male/female)	11/25	25/38	0.364
Follow-up (month)	11.2 ± 7.4	12.1 ± 9.4	0.451

**Table 2:** Clinical results from two groups.

	Group EI (n = 36)	Group LI (n = 63)	*p* Value
BMI (g/cm^2^)	23.3 ± 3.3	21.7 ± 3.7	0.101
Surgery duration (min)	30.2 ± 7.4	37.1 ± 8.9	0.0004 **
Final VAS (mm)	11.3 ± 10.9	13.5 ± 13.2	0.722
Revision surgery	3/36 (8.3%)	4/63 (6.3%)	0.711

** *p* < 0.01.

**Table 3:** Postoperative radiological results of two groups.

	Group EI (n = 36)	Group LI (n = 63)	*p* Value
Preop LKA (degree)	8.31 ± 6.48	9.37 ± 6.84	0.457
Postop LKA (degree)	6.41 ± 5.22	6.68 ± 5.28	0.726
Final LKA (degree)	7.47 ± 5.33	7.97 ± 6.10	0.827
Preop VKA (degree)	12.8 ± 7.38	14.9 ± 6.73	0.179
Postop VKA (degree)	5.72 ± 4.15	7.70 ± 5.01	0.100
Final VKA (degree)	9.61 ± 5.83	9.86 ± 6.05	0.870
Cement volume (mL)	7.42 ± 1.54	6.3 ± 1.93	0.007 **
Cement leakage	11 (30.1%)	12 (19.0%)	0.192
AVF	3 (8.3%)	21 (33.3%)	0.034 *
BMD (g/cm^2^)	−2.09 ± 0.96	−1.96 ± 1.41	0.911

*: *p* < 0.051, **: *p* < 0.01.

## Data Availability

The data presented in this study are available in the article.
